# HMG-CoA reductase expression in primary colorectal cancer correlates with favourable clinicopathological characteristics and an improved clinical outcome

**DOI:** 10.1186/1746-1596-9-78

**Published:** 2014-04-07

**Authors:** Erik Bengtsson, Pashtrik Nerjovaj, Sakarias Wangefjord, Björn Nodin, Jakob Eberhard, Mathias Uhlén, Signe Borgquist, Karin Jirström

**Affiliations:** 1 Department of Clinical Sciences, Oncology and Pathology, Lund University, 221 85 Lund, Sweden; 2 Science for Life Laboratory, Royal Institute of Technology, 171 21 Stockholm, Sweden; 3 School of Biotechnology, AlbaNova University Center, Royal Institute of Technology, 106 91 Stockholm, Sweden

**Keywords:** HMG-CoA reductase, Immunohistochemistry, Colorectal cancer, Prognosis

## Abstract

**Background:**

An association between tumor-specific HMG-CoA reductase (HMGCR) expression and good prognosis has previously been demonstrated in breast and ovarian cancer. In this study, the expression, clinicopathological correlates and prognostic value of HMGCR expression in colorectal cancer was examined.

**Findings:**

Immunohistochemical expression of HMGCR was assessed in tissue microarrays with primary tumours from 557 incident cases of colorectal cancer in the Malmö Diet and Cancer Study. Pearson’s Chi Square test was applied to explore the associations between HMGCR expression and clinicopathological factors and other investigative biomarkers. Kaplan Meier analysis and Cox proportional hazards modeling were used to assess the relationship between HMGCR expression and cancer-specific survival (CSS) according to negative vs positive HMGCR expression.

A total number of 535 (96.0%) tumours were suitable for analysis, of which 61 (11.4%) were HMGCR negative. Positive cytoplasmic HMGCR expression was associated with distant metastasis-free disease at diagnosis (p = 0.002), lack of vascular invasion (p = 0.043), microsatellite-instability (p = 0.033), expression of cyclin D1 (p = <0.001) and p21 (p = <0.001). Positive HMGCR expression was significantly associated with a prolonged CSS in unadjusted Cox regression analysis in the entire cohort (HR = 1.79; 95% CI 1.20-2.66) and in Stage III-IV disease (HR = 1.71; 95% CI 1.09-2.68), but not after adjustment for established clinicopathological parameters.

**Conclusions:**

Findings from this prospective cohort study demonstrate that HMGCR is differentially expressed in colorectal cancer and that positive expression is associated with favourable tumour characteristics and a prolonged survival in unadjusted analysis. The utility of HMGCR as a predictor of response to neoadjuvant or adjuvant statin treatment in colorectal cancer merits further study.

**Virtual slides:**

The virtual slides for this article can be found here: http://www.diagnosticpathology.diagnomx.eu/vs/2115647072103464.

## Findings

### Background

Colorectal cancer (CRC) is the third most common form of cancer in the world, with an estimated annual global incidence of more than 1.2 million new cases and around 600 000 deaths from the disease [1]. Although many efforts have been made to find molecular markers to identify high-risk disease and to select patients for adjuvant treatment, none have yet been implemented into routine clinical practice.

It has recently been demonstrated that elevated expression of the enzyme 3-hydroxy-3-methylglutharyl-coenzyme A reductase (HMGCR) correlates with favourable prognosis in breast and ovarian cancer [2,3], and with an improved response to tamoxifen [4] and pre-surgical statin treatment [5] in breast cancer. Several epidemiological studies have shown a link between use of statins, inhibitors of HMGCR, and a significantly reduced CRC incidence [6-8], and statins have also been demonstrated to exert anti-neoplastic properties in CRC cells *in vitro*[9,10].

To the best of our knowledge, the expression and prognostic significance of HMGCR in CRC has not yet been reported. The aim of this study was therefore to analyse tumour-specific expression of HMGCR by immunohistochemistry in tissue microarrays (TMAs) with primary tumour specimens from incident CRC cases diagnosed within a large, prospective cohort study, and to explore its relationship with established clinicopathological and tumor biological parameters, and survival.

### Methods

Until 31 Dec 2008, 626 incident cases of CRC had been registered in the Malmö Diet and Cancer Study (MDCS) [11-13]. Cases were identified from the Swedish Cancer Registry up until 31 Dec 2007, and from The Southern Swedish Regional Tumour Registry for the period of 1 Jan - 31 Dec 2008. Information on vital status and cause of death was obtained from the Swedish Cause of Death Registry up until 31 Dec 2009. Follow-up started at date of diagnosis and ended at the date of death, emigration or end of follow-up, whichever came first. All tumours with available slides or paraffin blocks were histopathologically re-evaluated on haematoxylin and eosin stained slides. Histopathological, clinical and treatment data were obtained from the clinical and/or pathology records. TNM staging was performed according to the American Joint Committee on Cancer (AJCC) [14]. Patient and tumour characteristics of the cohort have been described in detail previously [15-19]. Ethical permission was obtained from the Ethics Committee at Lund University (Ref. nr 51/90 and 530/2008).

TMAs were constructed as previously described using duplicate 1.00 mm cores [16].

For immunohistochemical analysis of HMGCR, 4 μm TMA-sections were automatically pre-treated using the PT-link system (DAKO, Glostrup, Denmark) and then stained in an Autostainer Plus (DAKO) with a polyclonal anti-HMGCR antibody HPA008338, Atlas Antibodies AB, Stockholm, Sweden diluted 1:150. This antibody has been validated (http://www.proteinatlas.org) and used in a recent window-of-opportunity statin trial in breast cancer [5].

HMGCR was expressed in the cytoplasm of the tumour cells, without distinct membranous staining as previously shown in e.g. breast cancer [2]. In line with previous studies, HMGCR was generally expressed in >50 percent of tumour cells in all positive cases [2,3,20]. Therefore, only the cytoplasmic staining intensity was taken into account, and denoted as 0 = >1% of cells being negative, 1 = weak expression in any fraction >1%, and 2 = moderate/strong expression in any fraction >1%. The staining was evaluated by three independent observers, who were blinded to clinical and outcome data (EB, PN and KJ). Scoring differences were discussed in order to reach consensus.

Immunohistochemical staining and evaluation of cyclin D1, p21, p27, p53, and beta-catenin expression has been described previously [15,17]. Immunohistochemical staining for microsatellite instability (MSI) screening status was performed as previously described, whereby tumours lacking expression of any of the DNA mismatch repair proteins MLH1, PMS2, MSH2 or MSH6 were denoted as having a positive MSI screening status, and tumours expressing all MMR proteins as being microsatellite stable (MSS) [18]. Analysis of KRAS and BRAF mutational status was performed by pyrosequencing as previously described [21,22]. Pearson’s Chi Square test was used to explore the associations of HMGCR expression with clinicopathological and investigative parameters. Kaplan-Meier analysis and log rank test were used to illustrate differences in cancer specific survival (CSS) according to negative vs positive HMGCR expression. Cox regression proportional hazards models were used for estimation of hazard ratios (HRs) for death from CRC and overall death in both uni- and multivariable analysis, the latter adjusted for age, sex, tumour (T)-stage, lymph node metastasis (N-stage), distant metastasis (M-stage), differentiation grade, vascular invasion and MSI status. A backward conditional selection method was used for variable selection by the model. All tests were two-sided. A p-value of 0.05 was considered significant. All statistical analyses were performed using IBM SPSS Statistics version 20.0.

### Results

**Figure 1:**
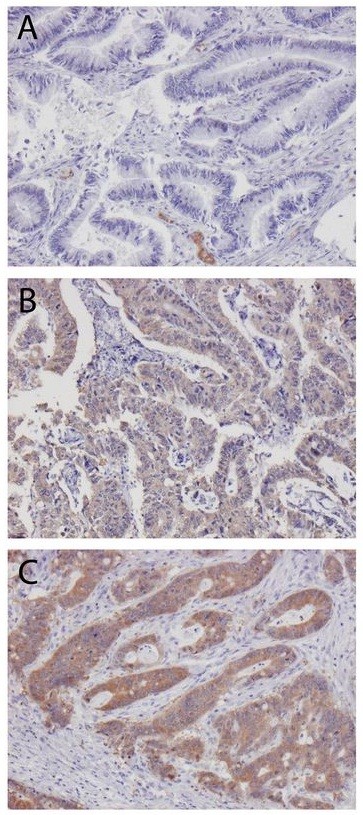
**HMG-CoA reductase expression in colorectal cancer.** Sample immuohistochemical images (10X) of primary colorectal cancer with **(A)** negative; **(B)** weak and **(C)** strong HMG-CoA reductase expression.

HMGCR expression could be evaluated in 535 (96.0%) cases, of which 61 (11.4%) were negative, 206 (38.5%) displayed weak and 268 (50.1%) displayed strong expression. Representative immunohistochemical images are shown in Figure 1. Normal colonic mucosa displays moderate-strong HMGCR expression (http://www.proteinatlas.org).

As shown in Table 1, there were significant associations between HMGCR expression and distant metastasis-free disease (p = 0.002), lack of vascular invasion (p = 0.043) and positive MSI screening status (p = 0.033). Moreover, HMGCR expression was significantly associated with expression of cyclin D1 (p < 0.001) and p21 (p < 0.001). There were no significant associations between HMGCR expression and age, sex, T-stage, N-stage, differentiation grade, mucinous histology, beta-catenin grades, p53 status, KRAS or BRAF mutation (data not shown).

**Table 1:** Significant associations of HMG-CoA reductase expression with clinicopathological characteristics and investigative factors

	**HMG-CoA reductase expression**		
**Factor**	**Negative**	**Positive**	**P-value**
**N(%)**	**61 (11.4)**	**474 (88.6)**	
**M stage**			
0	41(68.3)	395 (84.4)	0.002
1	19 (31.7)	73 (15.6)	
Missing	1	6	
**Vascular invasion**			
No	11 (32.4)	142 (50.7)	0.043
Yes	23 (67.6)	138 (49.3)	
Missing	17	194	
**Microsatellite instability**			
MSS	52(94.5)	371 (83.6)	0.033
MSI	3 (5.5)	73 (16.4)	
Missing	6	30	
**Cyclin D1 expression**			
Negative	22 (37.3)	80 (17.7)	<0.001
Positive	37 (62.7)	371 (82.3)	
Missing	2	23	
**p21 expression**			
Negative	20 (33.9)	53 (11.8)	<0.001
Positive	39 (66.1)	396 (88.2)	
missing	2	25	

The categories marked as missing were not included in the analysis.

**Figure 2:**
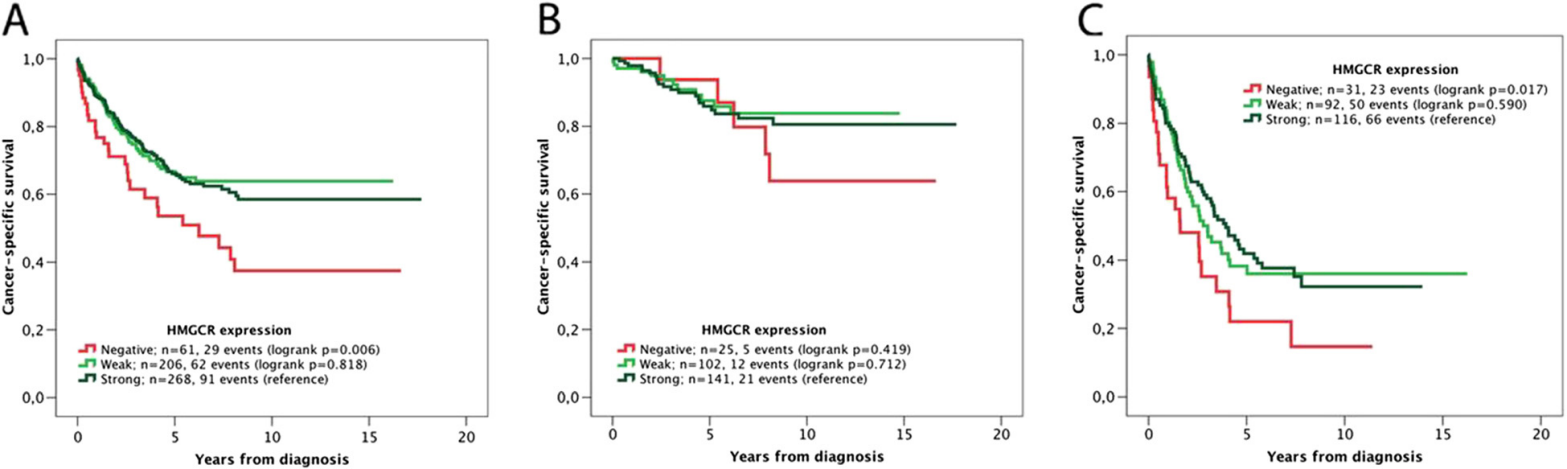
**Kaplan-Meier estimates of the prognostic impact of HMG-CoA reductase expression.** Kaplan Meier analysis and log rank test of colorectal cancer specific survival according to negative, weak and strong HMGCR expression in **(A)** all patients, **(B)** patients with stage I-II disease and **(C)** patients with stage III-IV disease.

Kaplan-Meier analysis and log rank test revealed a significant association between positive HMGCR expression and a prolonged CSS in the full cohort and in Stage III-IV (metastatic) disease, but not in Stage I-II (non-metastatic) disease (Figure 2). Notably, survival rates were similar for tumours with weak and moderate/strong expression (Figure 2).

Unadjusted Cox regression analysis confirmed the significant correlation between positive HMGCR expression and an improved CSS in the entire cohort (HR = 1.79; 95% CI 1.20-2.66) and in stage III-IV disease (HR = 1.71 (1.09-2.68) (Table 2). However, these associations did not remain significant in multivariable Cox regression analysis, adjusted for age, sex, T-stage, N-stage, M-stage, differentiation grade, vascular invasion and MSI status (Table 2). Similar associations were found using overall survival as endpoint (data not shown). The prognostic value of HMGCR did not differ by sex or tumour location (data not shown). There was no significant association between HMGCR expression and response to adjuvant treatment in curatively treated patients with stage III-IV disease (data not shown).

**Table 2:** Cox proportional hazards analysis of colorectal cancer specific survival according to HMG-CoA reductase expression

	**Full cohort**		**Stage I-II disease**		**Stage I-II disease**	
	**HR (95%CI)**	***n (events)***	**HR (95%CI)**	***n (events)***	**HR (95%CI)**	***n (events)***
	*Unadjusted*		*Unadjusted*		*Unadjusted*	
HMGCR pos	1.00	474 (153)	1,00	243 (33)	1,00	208 (116)
HMGCR neg	1.79 (1-20-2.66)	61 (29)	1.56 (0.61-4.01)	25 (5)	1.71 (1.09-2.68)	31 (23)
	*Adjusted*		*Adjusted*		*Adjusted*	
HMGCR pos	1.00	406 (126)	1.00	227 (30)	1.00	179 (96)
HMGCR neg	0.85 (0.51-1.41)	45 (19)	1.23 (0.41-3.70)	23 (4)	0.82 (0.46-1.46)	22 (15)

Multivariable analysis included adjustment for age (continuous), T-stage (1-2, 3, 4), sex, N-stage (0,1,2), M-stage, differentiation grade (high-intermediate vs. low), vascular invasion (no, yes, unknown) and MSI screening status.

### Discussion

The results from this comparatively large cohort study demonstrate that HMGCR expression in CRC is associated with clinically less advanced tumours and a more favourable prognosis. These findings are in line with previous studies on e.g. breast and ovarian cancer [2,3].

The correlation between HMGCR expression and a prolonged CSS did however not remain significant in adjusted analysis, and the lack of independent prognostic information for HMGCR expression may be explained by its significant association with established prognostic factors, in particular M-stage and vascular invasion. As the results in this study are entirely based on analysis of primary tumours, it would also be of interest to compare HMGCR expression in primary and metastatic lesions in future studies [23,24]. Moreover, as data on recurrent disease was not available for the patients in this cohort, future studies should also consider the association of HMGCR expression with to time to recurrence, preferably in cohorts where this information has been recorded prospectively.

The finding of an association between HMGCR expression and positive MSI screening status is of potential interest, as MSI tumours may be less responsive to adjuvant treatment with 5-fluorouracil-based chemotherapy [25-27]. Speculatively, CRC patients with MSI/HMCGR expressing tumours may instead benefit from adjuvant statin treatment, in particular as HMGCR expression has been demonstrated to predict an improved response to pre-surgical statin treatment in breast cancer [5]. Notably, in this cohort, positive MSI status has been demonstrated to be associated with a significantly improved prognosis in patients with stage III-IV disease, but not to be predictive of response to adjuvant chemotherapy [15]. Future studies should also address whether HMGCR expression in CRC is affected by different neoadjuvant treatment regimens [24,28].

Unfortunately, it was not possible to examine associations of HMGCR expression with statin use in this cohort, since the number of cases for whom statin medication was reported at baseline was too small [2]. However, a previous study demonstrated associations of high tumour-specific HMGCR expression in post-menopausal breast cancer with use of hormone replacement therapy and obesity [2]. These findings suggest that the influence of lifestyle factors and anthropometric measures on HMGCR expression in CRC also merits further study.

Statin-mediated blockade of the mevalonate pathway triggers a marked increase of inactive HMGCR in cultured cells [29] and statin treatment has also been demonstrated to induce cell cycle arrest and up-regulation of p21 and p27 in colorectal cancer *in vitro*[10]. In this context, the significant associations of HMGCR expression with expression of p21 and cyclin D1found here also indicate a potential cell-cycle regulatory role for HMGCR in colorectal cancer *in vivo*. Of note, high cyclin D1 expression has previously been found to correlate with good prognosis in this cohort, in particular in male CRC [17].

### Conclusions

This study provides a first report of tumour-specific HMGCR in colorectal cancer, and its association with favourable clinicopathological characteristics and an improved prognosis. The utility of HMGCR expression as a predictor of response to different neoadjuvant or adjuvant treatment regimens in colorectal cancer should be investigated in future studies.

## Abbreviations

CRC: Colorectal cancer; CSS: Cancer specific survival; HMGCR: 3-hydroxy-3-methylglutharylcoenzyme A reductase; HR: Hazard ratio; IHC: Immunohistochemistry; MDCS: Malmö Diet and Cancer Study; MSI: Microsatellite instability; MSS: Microsatellite stability; TMA: Tissue microarray.

## Competing interests

The authors declare that no competing interests exist.

## Authors’ contributions

EB and PN performed the immunohistochemical evaluation, statistical analyses and drafted the manuscript. BN carried out the IHC stainings and assisted with the collection of clinical data. SW and JE collected clinical, follow-up and treatment data. MU contributed to the conception and design of the study. SB conceived of the study and helped draft the manuscript. KJ conceived of the study, carried out the histopathological re-evaluation, evaluated the immunohistochemistry, and helped drafted the manuscript. All authors read and approved the final manuscript.
